# Per- and Polyfluoroalkyl Substances (PFAS): Significance and Considerations within the Regulatory Framework of the USA

**DOI:** 10.3390/ijerph182111142

**Published:** 2021-10-23

**Authors:** Blake Langenbach, Mark Wilson

**Affiliations:** Department of Environmental Health Sciences, Tulane University School of Public Health & Tropical Medicine, New Orleans, LA 70112, USA; blangenb@tulane.edu

**Keywords:** perfluoroalkyl, polyfluoroalkyl, PFAS, perfluorooctanesulfonic acid (PFOS), perfluorooctanoic acid (PFOA), hexafluoropropylene oxide-dimer acid (HFPO-DA), GenX, AFFFs, Chemours

## Abstract

Per- and polyfluoroalkyl substances (PFAS) are an emerging environmental crisis. Deemed *forever chemicals*, many congeners bioaccumulate and are incredibly persistent in the environment due to the presence of the strong carbon-fluorine covalent bonds. Notable PFAS compounds include perfluorooctanesulfonic acid (PFOS), perfluorooctanoic acid (PFOA), and GenX. Robust toxicological knowledge exists for these substances, but regulatory decisions based on this knowledge has fallen behind. The United States Environmental Protection Agency (EPA) has addressed this issue with the PFAS Action Plan and EPA Council on PFAS, but the regulatory framework is severely lacking. Currently, no federal regulations or standards exist. Many occupational and non-occupational human cohorts exist that can lend knowledge on the environmental implications of PFAS and associated health effects. Occupationally, firefighters face significant exposure risks due to use of PFAS containing aqueous film-forming foams (AFFFs) and personal protective equipment contamination. Non-occupationally, wastewater discharge in North Carolina led to chronic and widespread residential exposure to GenX via drinking water contamination. This public health review seeks to convey the current and future significance of PFAS as an environmental contaminate, to lend considerations on regulatory frameworks within the USA, and to help guide and promote the need for future epidemiological studies in order to tackle this environmental emergency. While the PFAS Action Plan creates a scientific and regulatory foundation, it is important to take these lessons and apply them to future environmental health issues.

## 1. Introduction

Per- and polyfluoroalkyl substances (PFAS) are a group of man-made organofluorine chemical compounds that contain multiple fluorine atoms attached to an alkyl chain with the moiety C_n_F_2n+1_—[1]. PFAS’ surfactant ability is much more effective in reducing the surface tension of water than other hydrocarbons, lending itself to be cost-effective and used in several industries worldwide. Notable PFAS compounds include perfluorooctanesulfonic acid (PFOS), perfluorooctanoic acid (PFOA), and GenX—a replacement for PFOA.

Strong carbon–fluoride bonds allow PFAS to have high thermal and chemical stability, making these substances incredibly resistant to both environmental and metabolic degradation [2]. With this, PFAS have been deemed as *forever chemicals*, as they are among the most environmentally persistent substances among organic chemicals [2]. Biomonitoring studies has shown that certain PFAS are ubiquitous throughout the U.S. population [3]. The primary routes of exposure are oral ingestion and dermal contact with contaminated environmental media, such as food, water, and dust, via use of commercial and industrial products. Human exposures occur in many occupational and non-occupational settings, most notably in manufacturing processes and contamination of groundwater and drinking water.

With more than 8000 different types of PFAS according to the Environmental Protection Agency’s (EPA) toxicity database, health effects have been shown in laboratory animal toxicology studies, human epidemiological studies, and occupational cohorts [2]. Areas of toxicological effects include detrimental impacts on development; the endocrine, reproductive, cardiovascular, hepatic, and immune systems; as well as cancer [4]. With this, the EPA has implemented a PFAS Action Plan and issued a 70-parts-per-trillion health advisory level for the sum concentrations of both PFOA and PFOS, but only few regulatory determinations have been imposed so far [5]. PFOA and PFOS have been the primary focus of epidemiological studies, toxicological data, and regulations due to the timing of their discoveries and past industrial usage, but other PFAS have been garnering more attention and emphasis.

With such an emerging public health and occupational health issue, there are several gaps in knowledge that must be addressed: toxicological mechanisms, hazard characterizations, regulatory frameworks, and creating longitudinal occupational and non-occupational cohorts to follow over time. With thousands of various congeners, understanding their environmental fate, transport, and potential for toxicity is critical in helping address these gaps. Leveraging modern resources, such as national and state biomonitoring programs and information sharing databases, is key in navigating prospective cohort studies, completing toxicological hazard profiles, and evaluating remediation processes. In doing so, this review seeks to show the worldwide significance for PFAS exposures as well as present recommendations in dealing with such a modern and future environmental emergency within the regulatory structure of the United States.

## 2. Historical Significance

The chemistry of PFAS was discovered in the late 1930s, and widespread usage in the United States began in the 1940s [5]. Soon, polyfluoroalkyl usage was popularized and began to be used in an array of commercial products and in widespread industrial applications. Commercial products range from cleaning and nonstick items, soil repellants, paints, coating, waxes, and fluoropolymers. Industrial processes include fluorotelomerization, electrochemical fluorination, oil recovery, electronic manufacturing, and chrome plating. One major industry is their role in creating aqueous film-forming foams (AFFFs), which effectively extinguishes fires involving highly flammable liquids [1]. However, this consequently presents a concerning and common occupational exposure scenario for firefighters and their use for this purpose results in environmental contamination.

PFAS was not identified as an emerging environmental and health concern until the early 2000s, when PFOS contamination was first shown in wildlife, even though internal reports and studies had identified the potential for toxicity and drinking water contamination decades earlier [6,7]. In 2001, researchers found concentrations of PFOS in wildlife tissue—species such as seals, polar bears, albatrosses, and more—on a global scale [6]. Samples were studied from a range of environments, ranging from the Mediterranean Sea and the Baltic Sea to the Great Lakes and Arctic oceans [6]. Results showed higher concentrations of PFOS in more industrialized and populated areas but also showed levels of PFOS in very remote marine areas [6]. This suggested that not only did PFOS bioaccumulate towards higher trophic levels but was also distributed globally [6,8]. Once published, questions arose about environmental persistence, toxicological effects, and the implications of bioaccumulation in humans.

As early as 1966, a fluorine biomonitoring study, which was designed to determine background levels of fluorine in the general population, identified exposure to fluorinated hydrocarbons. These findings indicated non-occupational exposures were occurring [9]. Other biomonitoring studies have since confirmed these findings. A study in 2001 confirmed that levels of PFOS and various analytes were detected in all 65 different human serum samples purchased from a biological supply company [10]. While levels of PFOA had been detected in the blood of fluorochemical industry workers decades prior, this study suggested human exposure via non-occupational routes [1]. Confirming this, one study conducted by the Centers of Disease Control and Prevention (CDC) found PFOA in almost all serum samples of over 2000 participants with a geometric mean serum level of 3.92 ng/mL [11]. Occupationally, one study from 2004–2005 showed that workers near a facility had an average serum PFOA level of 1000 ng/mL [12]. With these epidemiological studies, research shifted to a heavy focus on human exposure pathways, especially in non-occupational settings: food, groundwater, drinking water, airborne dust, breast milk, and so forth [1]. Studies showed ubiquitous, chronic exposure to background concentrations of PFAS across their lifetime, but toxicological information and regulatory decisions were missing. Nevertheless, persistence and bioaccumulation in both wildlife and humans has been clearly shown.

## 3. Toxicological Implications on Human Health

Mechanistic toxicity data regarding PFAS have been mostly confined to laboratory animal studies, but epidemiological studies involving occupational cohorts and certain non-occupationally exposed populations have led to a better understanding of potential exposure-related effects in humans.

The toxicokinetics of PFAS has been determined in laboratory animal studies and human cohorts. Systemic absorption can occur via oral, inhalation, and dermal exposure. Once exposed, PFAS is widely distributed throughout the body, accumulating mostly in the kidneys, liver, and blood since it can bind to albumin [13]. PFAS can be also transferred to nursing infants or the fetus during pregnancy [14,15]. Some studies have suggested that PFAS do not undergo chemical reactions and are therefore not metabolized or biotransformed [16,17], while others have published reports based on the measurement of PFAS metabolites in vivo [18]. Rates of elimination vary across the type of compound and animal species with differences in age and sex, but humans normally eliminate these substances through urine [19]. Some elimination half-lives include 72–81 h for PFBA, 2.1–8.5 years for PFOA, and 3.1–7.4 years for PFOS [20,21,22,23,24].

Evidence has shown that many PFAS have similar health outcomes, but there is also evidence that most of the compounds differ qualitatively and mechanistically [4,25]. Considering the thousands of poly- and perfluoroalkyls that exist, the Agency for Toxic Substances and Disease Registry (ATSDR) has conducted an extensive literature review of epidemiological studies involving PFOA, PFOS, and 12 other PFAS that suggest links between PFAS exposure and health outcomes in humans, published in PFAS’ ATSDR toxicological profile presented in Table 1.

Children are considered the most vulnerable population due to bioaccumulation, ability to transfer in utero, and possible PFAS-induced developmental effects from conception to adulthood. In one study involving over 12,000 children, higher PFOA and PFOS levels led to an increased risk of high cholesterol [26]. With immunotoxicity, many studies have shown suppressive antibody responses associated with higher serum levels of PFOA, PFOS, PFHxS, and PFDeA [27,28,29,30,31]. Increased asthma diagnosis was associated with higher PFOA serum levels [32]. One study involving children from ages 8 to 18 showed higher PFOA serum levels led to reduced odds of reaching puberty [33]. Despite these significant associations, other studies report non-significant effects.

Mixed studies have shown that higher PFOA or PFOS levels has been shown to decrease birth weight, but data are unclear [34,35]. Many studies have found no significant associations between certain parameters: PFOA and IQ, PFOA/PFOS and ADHD diagnosis, and PFOA and impulsivity [36,37,38]. The varying nature of PFAS mechanisms may explain the results of these epidemiological studies, as only certain substances are considered. Due to PFAS’ inherent properties and lack of toxicological data, children are still considered the most vulnerable population despite mixed associations. Currently, no epidemiological studies have examined populations with an inherent increased susceptibility to PFAS toxicity. However, those with pre-existing conditions, such as higher cholesterol levels or compromised liver functions, are more susceptible to PFAS’ health effects.

Detection methods for toxicity testing or exposure are based on biomarkers. The standard accepted biomarkers for PFAS exposure are based on serum measurement or whole blood perfluoroalkyl concentration [4]. When testing recent exposure, such as in occupational cohorts, elevated serum levels may not actually be indicative due to bioaccumulation properties and long half-lives of PFAS. One study with rats showed that hair could be a reliable biomarker, but a study in humans had mixed levels of detectability [39,40].

Data regarding chronic toxicity in humans are difficult to quantify. Epidemiological studies regarding chronic endpoints have difficulty in determining historic exposure, lifetime bioaccumulation, and compounded PFAS mixtures. The only ethical way to determine associations with chronic toxicity is through occupational cohorts or non-occupationally exposed populations followed for decades. Either way, correcting for prior risk factors or susceptibilities and determining specific PFAS health effects is difficult, especially with unknown levels of prior bioaccumulation before exposure. With this, most human epidemiological studies have analyzed serum levels of PFAS associated with increased risk of certain health outcomes to help guide certain associations. However, many of these associations are most likely from a mixture of serum PFAS, and health effects may not occur until later years.

While the ATSDR toxicological profile for PFAS was released in May of 2021, there are several knowledge gaps related to this chemical class. As a chemical class, the spectrum of PFAS consists of distinct compounds that have very different chemicals structures and properties with varying solubilities, reactivities, states of matter, and so forth [2]. With thousands of structurally unique PFAS, it is critical to treat these substances as a class of persistent chemicals in order to save resources and time instead of developing hazard and toxicological profiles for each one [2]. It is plausible to reason that within a class of chemical, despite certain unique chemical or physical properties, a highly persistent organic substance is likely to represent an exposure risk [2]. This approach has been used for polychlorinated biphenyls (PCBs), another class of persistent chemicals with significant exposure potential associated with developmental, endocrine, immunological, and cancer-influencing effects [41].

## 4. Regulatory Framework: EPA and ATSDR

### 4.1. Environmental Protection Agency (EPA): Framework

As of January 2021, the EPA has made significant strides in implementing their PFAS Action Plan, following their traditional risk assessment model by conducting large-scale, high-throughput in-vitro assays on 150 novel and emerging PFAS and testing for multiple endpoints, focusing on PFOA and PFOS specifically [5]. Attention was focused on these two substances because they were discovered very early, as PFAS have been the most used in industrial processes and pose the greatest, most common risk for human exposure. The EPA has published non-cancer oral reference doses (RfDs) at 0.00002–0.00015 mg/kg/day for both PFOA and PFOS based on rodent exposure studies, but no current federal regulations exist [5]. While progress is being made in updating the regulatory framework for PFAS, several limitations exist that require more attention and urgency on a federal level [5] (Table 2).

The EPA has yet to finalize regulatory standards or limits, only setting health advisory levels for PFOS and PFOA. Under the SDWA, lower limits should be imposed for all detectable PFAS until federal regulation can pass. Under the UCMR 5, innovative and precise detection methods need to be published in helping analyze and understand the nature of these substances. As of September 2021, the EPA has released an internally certified method for detecting 28 different PFAS specifically in oily matrices [42]. Under CERCLA, all detectable PFAS should be listed as hazardous substances and pollutants regardless of the circumstances. Under RCRA, guidelines for the remediation process must be expanded and refined. Under the CAA, inhalation exposure data have yet to be published by regulatory agencies, and standardized methods are lacking.

As of April 2021, the new EPA administrator established the EPA Council on PFAS to better understand, assess, and reduce the potential risks of PFAS to human and environmental health [43]. Building off the PFAS Action Plan, the council has been tasked with developing “PFAS 2021–2025—Safeguarding America’s Waters, Air, and Land”, charged with creating a multi-year strategy in delivering needed health protections to the general public, making its initial recommendations within 100 days of its creation. The council was charged with continuing close interagency coordination to assist and expand engagement opportunities with local communities, tribes, states, and federal partners [43]. Lastly, the council is expected to work with national programs and regions to maximize funding and financing impact, particularly focusing in leveraging state and federal funds to remediate PFAS in underserved and vulnerable communities [43].

President Biden’s American Jobs Plan includes upgrading and modernizing the nation’s drinking water, wastewater, and stormwater systems as well as tackling new contaminations and supporting clean water infrastructure in rural communities [44]. This includes $10 billion in funding to monitor and remediate PFAS in drinking water [44].

With more focus on PFAS by the EPA, ATSDR, and other governments, regulation action is finally coming together. However, thorough toxicity assessments should be conducted for major polyfluoroalkyl compounds. While in-vitro assessments yield insight, they are not effective in assessing the true health burden on animals or people. This in-vitro risk assessment paradigm also takes resources, energy, and time—too much time to conduct, publish, and turn data into federal regulations. Regulating these compounds in a proactive, conservative approach is health protective for environmental, occupational, and human health. The PFAS Action Plan and EPA Council is a sign of prioritizing this issue.

### 4.2. Agency for Toxic Substances and Disease Registry (ATSDR): Framework

The ATSDR has listed provisional oral minimal risk levels and human equivalent doses for a few polyfluoroalkyl substances Table 3. Provisional MRLs are based on lowest observable adverse effect levels (LOAEL) and no observable adverse effect levels (NOAEL) in intermediate duration laboratory animal studies [4]. Regulations for occupational health and industrial hygiene are lacking.

For PFOA and PFOS, the most sensitive target organs and pathways in laboratory animals were developmental, immunological, hepatic, and cancer endpoints [45,46,47,48,49,50,51,52,53,54,55].

For PFHxS, health outcomes in laboratory animals were largely hepatic and thyroid endpoints [56,57].

For PFNA, developmental and body weight results were prominent endpoints in laboratory studies [58,59].

Inhalation data are available for both PFOA and PFNA, but both the ATSDR and EPA have deemed the data are inadequate enough for deriving inhalation MRLs [4]. The Occupational Safety and Health Administration (OSHA) and the American Conference of Governmental Industrial Hygiene (ACGIH) has also not published any data regarding inhalation exposure due to limited endpoint data, difficulty in establishing a dose-response relationship, and lack of a standardized method for PFAS air sampling. The ACGIH has only classified PFOA as carcinogenic to animals with unknown relevance to humans [4].

Regarding airborne exposure, a recent study at a fluoropolymer manufacturing facility involving 26 explicit PFAS, including GenX, were added to a Community Multiscale Air Quality (CMAQ) model, the first air-modeling study for these substances [60]. The CMAQ model predicted 5% by mass of total emitted PFAS, and 2.5% of total GenX are deposited with 150 km of the plant [60].

### 4.3. Limitations & Recommendations

While the EPA is making great strides with the PFAS Action Plan, several limitations must be addressed. First, while all these standards focus on the main compounds PFOA and PFOS, the scope of the standards are limited. Thousands of PFAS congeners exist, so it is critical to develop detection methods that can analyze entire subclasses of PFAS, such as alkyl acids, ether acids, and fluoropolymer precursors, rather than specific compounds and analytes [61]. Secondly, the existing method limit of detection of 40 parts per trillion is high considering the national health advisory level of 70 parts-per-trillion. Federal guidelines should recommend screening levels as low as 1 part per trillion to ensure RCRA compliance and to be health conservative. Third, there are currently no established standardized method for characterizing PFAS in air samples. With PFAS being prevalent in industrial settings, air quality levels should also be prioritized in high-temperature emissions and their surrounding areas. Utilizing the recent CMAQ model in predicting atmospheric transport and fate of PFAS around fluoropolymer manufacturing sites can help with risk and exposure mapping both occupationally and non-occupationally. Fourth, and most importantly, inadequate water sampling has led to limited exposure assessments. With this, testing coverage is also limited, which leads to decreased exposure mapping and characterization of risk.

Under the SDWA, the health advisory limit should be lowered and include more PFAS chemicals. Under the CAA, other agencies should help prioritize air sampling methods and refine inhalation exposure doses. Regarding CERCLA/RCRA, several issues arise in the remediation process. Guidelines on how to remedy or reverse contamination are lacking, and some scenarios are effectively irreversible. With such low concentrations, the cost and energy in doing so is intensive. Paired with limited detection methods, remediation is also technically challenging. Therefore, regulations should establish concentration levels, breadth of exposure, and general protocols in determining PFAS hotspots.

The call to action in regulating these persistent chemicals has come two decades later after the discovery of PFAS being a globally distributed contaminant. Federally, attention towards these compounds has been lacking. Regulation has been slow, and corporations have taken advantage of certain mandates. Federal action is sometimes clouded with current administrative politics or corporate influence, hence the PFBS toxicity assessment breaching scientific integrity. However, it is crucial to act on breakthrough scientific research and form health-protective policies than compromise human health through slow regulation and federal inaction. Understanding this importance, several states have taken it upon themselves to enforce stricter mandates and regularly test drinking water for compliance.

With these current regulations and limitations in mind, this review seeks to highlight two key issues: occupational cohorts involving the usage of aqueous film-forming foams (AFFFs) and the recent non-occupational human exposure incident of GenX contamination in North Carolina drinking water. Analysis of these events intend to raise questions and recommend solutions to PFAS in the lens of environmental science and industrial hygiene.

## 5. Occupational Cohorts, Firefighters, and Aqueous Film-Forming Foam (AFFF)

PFAS’ exceptional surfactant ability and the thousands of possible congeners that exist lends itself to be involved in many fluoropolymer industrial processes. As a result of this, many occupational cohorts exist, each with completely different occupations, manufacturing processes, and exposure scenarios. Scenarios can be similar in how workers are exposed often through dermal contact or inhalation of dust and aerosols, but the PFAS congeners that are produced and their varying levels are often different per industrial process.

In a fluorochemical manufacturing plant in China, plasma samples were collected from 40 occupational workers and 52 control subjects in a city 80 km away from the factory in 2017 [62]. Mass spectrometry and metabolomic analysis detected 13 PFAS congeners, with six of them—PFBA, PFOA, PFBS, PFHxS, PFOS, and 6:2 Cl-PFESA—having significant concentrations. Geometric mean concentrations of the sum of these six congeners were 1700 ng/mL in occupational workers and 22.2 ng/mL in the control subjects, highlighting a stark difference in exposure scenarios. Of the potential biomarkers analyzed, 14 of them can be associated with fatty acid β-oxidation disorder, oxidative stress, and kidney injury [62].

Professional ski waxers are exposed to PFAS during waxing. In one occupational study analyzing whole blood, air, and aerosol samples, 13 perfluorocarboxylic acids, 4 perfluorosulfonic acids, 3 fluorotelomer alcohols, 3 fluorotelomer acids, and 3 unsaturated fluorotelomer acids were detected [63]. In technicians’ whole blood samples, PFOA, PFNA, perfluorohexadecanoic acid (PFHxDA), and perfluorooctadecanoic acid (PFOcDA) were all elevated. Several other metabolites were also detected, such as 5:3 and 7:3 fluorotelomer acid. In 37% of personal measurements, the occupational exposure limit of 2 mg/m^3^ was exceeded, with one aerosol concentration at 15 mg/m^3^ [63].

Firefighters also face a great risk of PFAS exposure with AFFF usage. AFFFs have been used by firefighters since the 1960s in extinguishing chemical solvent and hydrocarbon-fueled fires [64,65]. Its surfactant nature allows the fire to be coated and help prevent any oxygen interacting with fire [55]. The foam is usually a mixture of PFAS and other solvents that help act as anti-freezing agents [55]. Extensive usage of AFFF and the persistent nature of PFAS has led to severe environmental contamination; for instance, the Department of Defense has identified almost 700 millitary installations, which will require remediation [66].This occupational exposure scenario is unique in the fact that AFFF has the potential to create polyfluoroalkyl reservoirs in soil and groundwater, ultimately contributing to non-occupational exposure scenarios as well.

### 5.1. Occupational Cohort: Firefighters

During fire suppression, firefighters are most likely exposed via inhalation and dermal routes [55]. Firefighting suits that are contaminated also increase the risk of an oral exposure route via hand-to-mouth, eventually leading to the gastrointestinal tract [55]. Their personal protective equipment is actually made with PFAS-treated fluoropolymer textiles for water resistance [67]. Over time, PFAS from the outer shell ultimately migrates to the inner thermal layer, representing a possible dermal exposure [67]. Widespread foam usage during training exercises held on military bases and airports and during actual fires has led to widespread PFAS contamination into surface water, groundwater, soil, and sediment [64]. Firefighters suffer from a disproportionately high rate of various cancers, such as prostate and testicular cancer, that have also been linked to PFAS exposure [67]. However, due to the nature of their occupation, it is difficult to link specific health effects to PFAS exposure alone.

One aircraft accident simulation study in Finland studied eight male firefighters engaged in three consecutive training sessions over a course of three months [55]. Each simulation last about an hour and used Sthamex 3%, with PFOS and PFOA compounds being most prevalent at 240 μg/mL and 21 μg/mL, respectively [55]. Blood sampling was conducted four times and then analyzed via liquid chromatography and mass spectrometry [55]. Total serum PFAS concentrations between the firefighters ranged from 6.5 to 51 ng/mL [55]. These values range from double to thirteen times the general population’s geometric mean serum level of PFOA at 3.92 ng/mL [9]. Serum concentration is dependent on the amount, duration, proximity, and type of foam used as well as possible oral exposure from suit contamination. Though these documented exposures are concerning, potential human health effects at these levels are still poorly understood and long-term effects unknown.

In one study analyzing PFAS in fire station dust, 24 PFAS were measured in multiple rooms of 15 fire stations in Massachusetts, with many of them not using PFAS-containing AFFF at all [68]. When compared to station living rooms, the locker rooms for turnout gear had significant dust levels of total fluorine, PFHxA, perfluoroheptanoate (PFHpA), and perfluorodecanoate (PFDoDA). The predominant PFAS measured in the living rooms was N-ethyl perfluorooctane sulfonamidoacetic acid (N-MeFOSAA), which is a precursor to PFOS. With PFOS being phased out and some stations not using PFAS-containing AFFF, this precursor still persists [68].

With these two studies, it is evident that not only can firefighters be exposed to PFAS through intentional activities of quenching fires with AFFF but also through unintentional activities in the station and turnout gear. These exposures are primarily inhalation and dermal routes, but AFFF usage also creates environmental contamination that contributes to unintentional non-occupational exposure via soil and water.

### 5.2. AFFF Contamination in Soil & Groundwater

In one study analyzing the extent of AFFF contamination, 10 active United States Air Force (USAF) stations were selected with 40 samples taken and classified in low-, medium-, or high-AFFF release, representing emergency response, hangars and buildings, and testing, respectively [69]. Various sample matrixes were taken—surface and subsurface soil, sediment, surface water, and groundwater. Sixteen PFASs were detected, and across all sites and mediums, PFOS and PFHxS were the most prominent polyfluoroalkyls detected. ANOVA analysis showed a significant association between site classification of AFFF usage and mean PFAS concentration, suggesting facilities with higher AFFF usage, such as firefighting testing and training facilities, will have higher PFAS deposition. Regarding environmental contamination, it is evident that polyfluoroalkyls can be deposited through many different mediums [69].

In analyzing the extent of contamination via AFFF usage, one study collected 44 soil cores and 17 groundwater samples from a firefighter training area used since 1987 [64]. Samples were analyzed via solid phase extraction, liquid chromatography, mass spectrometer, and the total oxidizable precursor assay [64]. The most prevalent compounds were PFOS, fluorotelomer sulfonic acid (FTSA), and fluorotelomer sulfonamide alkylbetaine (FTAB) [64]. In soil samples measured 15 m below the surface past layers of clay, the highest total concentrations recorded were up to 357 μg/g in two areas considered possible entry points into aquifers [64]. Highest PFAS concentrations recorded ranged from 300 to 8300 ng/L in monitoring wells located around the training site and in the spring located towards the flow direction of groundwater [64]. With this, data indicates 30 years of AFFF usage have created a deep, long-term reservoir [64]. A lack of analytical standards for fluorotelomers and polymers, even with the oxidable precursor assay, resulted in unknown recovery percentages of the unidentified compounds collected and extracted and thus underestimated [64]. As the area of sampling is limited, the environmental impact from site contamination is also most likely underestimated [64].

Considering the vast number of USAF bases and airports using AFFF across several decades, PFAS reservoirs may be greatly underestimated. This creates potential non-occupational exposures as these underground reservoirs have the ability to seep into aquifers and drinking water sources. With this, exposure mapping is limited. State water quality and environmental departments should conduct regular sampling and soil monitoring with known or possible AFFF-contaminated areas, using the landfill leachate monitoring requirement sunder RCRA as a foundational framework. Levels reported with current sampling techniques may be underestimated due to PFAS mixtures, limited detection methods, and confined area sampling. However, assessing the extent of environmental impact can guide exposure mapping and remediation processes.

### 5.3. Safety Standards

Understanding its environmental impact, manufacturers of PFAS have started favoring shorter-chained fluorotelomers in producing AFFFs [55]. As of November 2020, 26 states have either proposed or passed regulations for PFAS in AFFF [70]. In 2019, Congress passed a law that phases out AFFF usage at all military sites by 2024 [70]. Most of these statutes limit or prohibit AFFF in training exercises, allows state programs to purchase and dispose of the foams, and requires reporting when the foam is used continuously [70]. As of July 2020, the European Commission Regulation has issued firefighting foam to not include concentrations greater or equal to 25 ppb of PFOA or its salt and 1000 ppb of one or combination of PFOA-related substance [71].

Fluorine-free foams have been available for decades, but their effectiveness has been debated. One of these foams, “Bio Ex Ecopol 3 × 3//3 × 6”, has shown to be similarly effective without contamination issues in Finland [65]. Worldwide, PFAS-free foams are being used by the Norwegian and Danish armed forces, all 27 major Australian airports, in major international airports like London Heathrow, and by oil and chemical manufacturers, like BP and Pfizer [71].

However, the United States Department of Defense (DOD) has stated that these PFAS-free foams that are commercially available do not meet the department’s safety standards in putting out aircraft, industrial, or structural fires quickly enough [72]. In finding a suitable replacement, research has focused on development, performance in large-scale capacity, and ecotoxicology [72].

Moving forward with the United States’ phaseout of fluorine based AFFF by 2024, industry and technology efforts must expedite suitable replacements. Fluorine-free foams must meet criteria while proving sufficient quenching capability without the caveat of environmental persistence. The remaining 24 states must also be pressed to limit AFFF usage in training exercises and require reporting its usage, but this should come as a federal mandate. Lastly, firefighter suits should also be redesigned by replacing PFAS-treated fluoropolymer textiles, but this also requires industrial innovation of new personal protective equipment.

In all, it is difficult to link the health effects of firefighters purely to PFAS due to the many occupational hazards and exposure scenarios they face, but the environmental contamination resulting from AFFF usage remains a serious, underrepresented issue and presents potential non-occupational exposure routes.

## 6. GenX Chemicals in North Carolina and Regulatory Limitations

Hexafluoropropylene oxide-dimer acid (HFPO-DA) and its ammonium salt, also known as GenX chemicals, was considered a safer substitute to ammonium perfluorooctanoate, the ammonium salt of PFOA, by the EPA [73]. GenX is commonly used in producing fluoropolymers as a safer alternative to PFOA [73]. The EPA is currently working on finalizing the toxicity assessment of these chemicals [74].

### 6.1. Chemours Facility

In 2009, the EPA stipulated a Consent Order for the production and use of GenX, requiring that at least 99% of GenX be captured in both air emissions and wastewater discharges [73]. In Fayetteville, North Carolina, the Chemours Facility produced GenX and stated that all wastewater from the plant would be capture and shipped off-site for disposal purposes [73]. However, in 2015, GenX was identified downstream from the facility in their wastewater discharge [75]. In 2016, 90 miles away downstream, concentrations at a drinking water intake were found up to 4500 ng/L—almost 100 times greater than EPA’s HAL [76]. GenX was subsequently reported in the drinking water surrounding the Cape Fear River, affecting more than 200,000 residents [73]. The state was able to stop the Chemours facility from releasing any more GenX in their wastewater, and drinking water concentrations have since declined enough to meet the state’s health goals [73]. However, it was determined that the requirements of the EPA’s Consent Order were not applicable if GenX and/or HFPO-DA was formed as by-products [73]. Currently, there is a court order against the facility by North Carolina’s Department of Environmental Quality.

### 6.2. EPA’s GenX Draft Toxicity Assessment

According to the draft toxicity assessment by the EPA, degradation data suggest these chemicals to be persistent—defined as a half-life longer than six months—in air, soil, water, and sediment [74]. GenX chemicals are expected to run off into surface water and leach to groundwater. Conventional wastewater or drinking water treatment is not expected to remove these chemicals. When released into the air or volatilized from water, these chemicals are expected to be stable, and long-range transport is possible. Data suggest these chemicals are more likely to remain in water with little partitioning to oil or sediment given their high water solubility. GenX chemicals are also highly resistant to biodegradation [74].

Most toxicological research towards polyfluoroalkyl substances have been focused on PFOA and PFOS. Currently, there are no human epidemiological studies for GenX chemicals. However, there are many studies involving various exposure pathways grouped into acute, subchronic, and chronic toxicity in rats and mice published in the draft toxicity assessment [74]. The EPA has stated that there is “suggestive evidence of carcinogenic potential” for oral exposure to GenX chemicals in humans, but there is no data available for dermal or inhalation exposure. Hazard summaries for GenX chemicals and their relevant toxicity endpoints are summarized below in Table 4.

Animal studies testing chemical toxicity are the gold standard and form the foundation of modern toxicology research to ensure human health and safety, often being implicated in regulatory decisions. GenX chemicals have a limited number of animal studies that show various toxicity endpoints. Due to its ability to persist and bioaccumulate, chronic endpoints are most relevant to human epidemiology and realistic exposure scenarios. Safety factors for humans are applied when using animal model data to imply human health effects, but this is not sufficient in regulatory decisions. It may be reasonable and expected to apply relevant toxicity data from PFOA to GenX to fill in necessary human toxicological endpoints and inform regulation. However, per the EPA, this does not correlate nor equate enough. This reasoning ultimately formed the basis of EPA’s decision to suggest GenX as a safer alternative at the time, which has now been shown to be just as problematic as PFOA to both wildlife and humans. In being health protective, the EPA should have conducted the necessary toxicological studies needed and infer endpoints from PFOA studies before recommending it as an alternative despite the gaps in knowledge and regulatory guidance.

### 6.3. Non-Occupational Cohort: GenX Exposure

North Carolina’s non-occupational cohort of GenX exposure to more than 200,000 residents can help inform our understanding of both acute and chronic GenX toxicity. However, only one biomonitoring study has been conducted since the incident. In 2018, the North Carolina Department of Health and Human Services (NCDHHS), partnered with the CDC and ATSDR, took biological samples from 30 participants in Bladen County and tested for 17 PFAS, including GenX [77]. Half of the participants were male, 25 were adults, average GenX concentration in their private wells was 680 ppt, and all of them used bottled water as their main source of drinking water [77]. Results showed that GenX was not detected in blood or urine, only one PFAS was detected in urine, nine of the 17 PFAS were detected in blood samples, and the median levels of PFHxS and n-PFOS detected were higher than median levels in the general U.S. population [77]. Despite these results, the exposure investigation seems limited: PFAS concentrations are only representative of the sampling time, sampling area is limited to one county, sampling population is quite small and similar, and the participants’ drinking water source [77]. Furthermore, many of the chemicals detected in biospecimens have no defined regulatory limits, which forces public health agencies to rely on assumptions about chemically induced health risk.

GenX contaminated wastewater and drinking water in the area, so testing participants whose primary drinking source is from water bottles does not seem indicative to real-time exposure or accurately represent the population of 200,000 people affected. Sampling area is confined to one county, whereas contamination spanned multiple counties and residential areas. With such heavy limitations, this biomonitoring study seems to only benefit the NCDHHS in being able to publish an exposure investigation into the incident that helped ease the public’s fear of GenX and trust in the state’s response. While this investigation was a start, it would be incredibly beneficial to North Carolina residents to follow through with widespread biomonitoring studies and to the toxicological profile if associations between GenX serum levels and human health effects were found. As unknown as GenX actually is, so are the issues of its environmental persistence and chronic health effects.

Further toxicological and ecological testing is needed when regulating chemicals, especially those that are considered replacements or substitutes for known bad actors. With a very similar chemical structure compared to PFOA and other PFAS, it should have been clear that GenX would likely cause similar toxic effects. More scientific insight should have been utilized into this regulatory decision by the EPA––lacking necessary toxicological data, hazard profile, and implications from PFAS suggest GenX not be considered as a suitable replacement for PFOA. However, recommending it as a substitute was most likely based on the fact that GenX was not yet considered an emerging neurotoxicant due to its incomplete profile.

## 7. Modern Solutions and Resources

Though the ubiquitous distribution of PFAS has been known for several decades, federal regulation has clearly been slow, primarily due to incomplete toxicology profiles and not understanding the true burden associated with PFAS exposure in relation to socioeconomics and healthcare. In tackling this environmental emergency, creating innovative solutions and leveraging modern resources are needed.

### 7.1. Cost-Benefit Analyses

Conducting cost-benefit analyses examining the socioeconomic and healthcare burden of PFAS exposure may assist regulatory makers and the public in understanding the global scope and urgency of this issue. In 2019, a published socioeconomic analysis of environmental and health impacts of PFAS exposure found that annual health-related costs ranged €2.8–4.6 billion for Nordic countries and €52–84 billion for all European Economic Area (EEA) countries [78]. Non-health related costs were estimated at €46 million–11 billion for Nordic countries [78]. Accounting for population size and exchange rates, the equivalent health-related annual costs in the United States would be $37–59 billion [79]. It is important to note these costs do not take into account lost wages, reduced quality of life, lost years of life, impacts on family and community, and many more social costs. In addition, costs are not paid via the polluter-pays principle but by health care providers, taxpayers, and ordinary people [79]. With this, cost-benefit analyses based in the United States are desperately needed to both quantify impact and guide policy processes.

Current methods in reducing PFAS levels in drinking water include reverse osmosis, ion exchange, dilution, or granular activated charcoal treatment—none of them fully eliminating these compounds. Wastewater treatment plants remove solids and pathogens, so polyfluoroalkyl substances coming into the plant are often discharged into receiving waters or sewage sludge. Municipalities can buy water from other distributors or implement new methods, but capital and infrastructure costs are significant [79]. For example, Orange County in California estimates $1 billion is needed to meet state recommended levels [79].

Costs related to the socioeconomics and healthcare burden, remediation, and drinking water and wastewater treatment for PFAS is significant and will most likely disproportionately affect vulnerable communities [79]. Failing to take timely action in regulation will only exacerbate costs and these burdens throughout generations. With this, performing cost-benefit analyses focused on the United States and creating innovative scientific methods in remediation is needed.

### 7.2. Network Building and Information Sharing

Leveraging modern resources with national health programs by biomonitoring exposed or potentially exposed populations can help inform spatial exposure mapping and toxicological data. As seen with the NCDHHS biomonitoring study, national programs and agencies are needed in helping keep scientific integrity intact by limiting political or social bias.

Nationally, the Association of Public Health Laboratories (APHL) has created a National Biomonitoring Network and the CDC has partnered with ATSDR to create PFAS Exposure Assessment Technical Tools to help health departments in all levels—state, local, regional, tribal—to conduct biomonitoring activities [80]. Six states have created their own PFAS biomonitoring programs, funded by the CDC [81]. With this, implementing and utilizing these health programs takes initiative and prioritization by state officials. Standardized guidelines and funding on a federal level would ultimately promote these actions. These health programs can then form networks between state and national organizations in information sharing.

Epidemiological and toxicological data shared between state and federal networks lends itself to refining health program capabilities as well as informatics. The power of trans-disciplinary data integration lends itself to better understanding PFAS distribution, socioeconomic effects, and exposure mapping. Computational systems could pool biomonitoring and cohort studies’ data to infer human health effects and form associations; help refine detection and remediation processes; and map possible PFAS-contaminated areas, AFFF reservoirs, and industrial incidents. However, a computational system dedicated to PFAS is lacking and may be limited by analytical capability and computing power. With the amount of national information and federal attention towards PFAS, an emerging computational system is currently needed. States enacting biomonitoring health programs can form the foundations of PFAS informatics, with federally funded organizations leading the charge in innovative computing power. State datasets can then be aggregated to form the basis of a national database.

## 8. Conclusions

Two decades after being publicly identified as a ubiquitously persistent and accumulating class of chemicals, the determination of toxicological mechanisms, hazard characterization, and regulatory frameworks enveloping PFAS have fallen behind. Mixed data due to unknown bioaccumulation and PFAS mixtures have clouded its toxicological profile. Regulating the entire class of PFAS and not just specific compounds is critical in being health protective and preventing future accidents [61].

While AFFF is being phased out by 2024, discovering a suitable, effective replacement for AFFF still remains a challenge. State water quality and environmental health departments need to regularly monitor soil and groundwater in AFFF-contaminated areas during its phaseout. Innovative solutions and detection methods are needed in these remediation and treatment processes.

Properly screening chemicals like GenX before approval is necessary. The full hazard assessment of perfluorooctane sulfonate (PFOS) and its salts released by the OECD is a good example framework of the level of assessment needed for new formulations of chemicals with a potential for toxicity [82]. In the absence of toxicological data for specific new or unevaluated chemicals, it is common for decisions to be made based on application of the precautionary principle. This requires assumptions, such as that the new or unevaluated chemical is equipotent or equitoxic or possesses some degree of relative potency compared to a known chemical with similar properties.

Conducting cost-benefit analyses in the United States can capture the true socioeconomic burden that PFAS places on healthcare systems, in occupational settings, and around vulnerable communities. Allocating state and federal funding in upgrading current water systems may be expensive but can drastically relieve these strains overtime. Better understanding these impacts can help guide policymakers and warrant regulation.

State organizations should leverage resources to conduct population biomonitoring around heavily PFAS-polluted areas and have follow-ups for several years to better understand toxicological implications and hazard characterization. Network building between governmental and non-governmental organizations can lend trans-disciplinary data integration that can also help guide policymakers and needed regulation.

In reality, PFAS toxicology in terms of impacts on human health is still shrouded in mystery. The thousands of compounds that exist cannot all be tested, but analyzing the main compounds––PFOS and PFOA––can form the foundations and allow for toxicological implications and hazard characterizations to be inferred for other polyfluoroalkyl chained compounds with the development of toxic equivalency factors (TEFs) or relative potency factors (RPFs). A similar approach has been taken for the evaluation of polycyclic aromatic hydrocarbons (PAHs), polychlorinated biphenyls (PCBs), and dioxins. Leveraging modern resources in biomonitoring programs and computational informatics can help complete these hazard profiles tremendously. With this, regulatory agencies must start being proactive instead of reactive with all environmental emergencies. In doing so, the country can enact more conservative and health-protective measures that safeguard both the environment and human health.

## Figures and Tables

**Table 1:** ATSDR General Health Outcomes and Effects among PFOA, PFOS, and 12 other PFAS.

Effects	Health Outcomes [4]
Hepatic:	Increased serum enzymes, decreased serum bilirubin
Cardiovascular:	Pregnancy-induced hypertension and/or pre-eclampsia
Endocrine:	Increased risk of thyroid disease
Immune:	Increased risk of asthma diagnosis, decreased antibody response to vaccine
Reproductive:	Increased risk of decreased fertility
Developmental:	Small decreases in birth weight

Adapted from ATSDR 2018 [4].

**Table 2:** Summary of U.S. Environmental Protection Agency’s (EPA) Regulatory Framework [5].

EPA Regulatory Framework	Summary of Regulation [5]
PFOA Stewardship Program (2006)	8 leading North American companies achieved a 95% reduction of PFOA + PFOA-related chemicals by 2015
Safe Drinking Water Act (SDWA)	No established maximum contaminant levels (MCLs) National health advisory level of 70 ng/L for sum concentration of PFOS + PFOA
Fifth Unregulated Contaminated Monitoring Rule (UCMR 5)	Requires sampling of lithium + 29 various PFAS between 2023 and 2025 EPA Method 533 for PFAS carbon chains of 4 to 12
Toxic Substances Control Act (TSCA)	294 PFAS reviewed, 191 regulated through orders and Significant New Use Rules (SNURs) Proposed SNUR that requires notifying EPA at least 90 days before usage of PFOA and related chemicals
Comprehensive Environmental Response, Compensation, and Liability Act (CERCLA)	PFAS currently not listed as hazardous substances PFOA and PFOS may be considered as pollutants or contaminated under certain circumstances
Resource Conservation and Recovery Act (RCRA)	Primary remediation goal of 70 parts per trillion Interim recommendation of 40 parts per trillion screening level for PFOA and/or PFOS Cleanup guidelines not fully established
Clean Air Act (CAA)	No published non-cancer reference concentrations for inhalation exposure for PFOS or PFOA No standardized analytical methods for PFAS in air
Toxicity Assessments	Perfluorobutane Sulfonic Acid (PFBS) assessment not published due to political interference and scientific integrity violations GenX assessment finalized in 2020

**Table 3:** ATSDR’s Oral Minimal Risk Levels (MRLs) and Human Equivalent Doses for Various PFAS.

PFAS	Oral MRL (mg/kg/day)	Human Equivalent Dose (mg/kg/day)
PFOA	3 × 10^−6^	0.00821 at the LOAEL
PFOS	2 × 10^−6^	0.000515 at the NOAEL
PFHxS	2 × 10^−5^	0.0047 at the NOAEL
PFNA	3 × 10^−6^	0.001 at the NOAEL

Adapted from ATSDR (2018) [4].

**Table 4:** Hazard Summary of GenX Chemicals in Rat and Mice Studies.

Effects	Health Outcomes
Hepatic:	Hepatocellular hypertrophy, increased liver-to-bodyweight ratio
Hematological:	Decreases in red blood cell count, hemoglobin, and red blood cell percentage in blood
Renal:	Kidney hypertrophy and blood urea nitrogen in female rats
Developmental/ Reproductive:	Decreased mouse pup weight, delays in balanopreputial separation and vaginal patency
Immunological:	Decreased serum globulin levels and spleen weight in female mice
Cancer:	Increased incidence of liver tumors (females) and combined pancreatic acinar adenomas and carcinomas (males)

Adapted from USEPA (2018) [74].

## Data Availability

No new data were created or analyzed in this study. Data sharing is not applicable to this article.
